# Novel Potential Biomarkers Associated With Epithelial to Mesenchymal Transition and Bladder Cancer Prognosis Identified by Integrated Bioinformatic Analysis

**DOI:** 10.3389/fonc.2020.00931

**Published:** 2020-06-30

**Authors:** Chengyuan Wang, Yujing Yang, Lei Yin, Ningde Wei, Ting Hong, Zuyu Sun, Jiaxi Yao, Zhi Li, Tao Liu

**Affiliations:** ^1^Department of Urology, The First Affiliated Hospital of China Medical University, Shenyang, China; ^2^Department of Medical Oncology, The First Affiliated Hospital of China Medical University, Shenyang, China

**Keywords:** bladder cancer (BC), weighted co-expression network construction (WGCNA), epithelial to mesenchymal transition (EMT), CORO1C, TMPRSS4

## Abstract

Bladder cancer (BC) is one of the most common malignancies in terms of incidence and recurrence worldwide. The aim of this study was to identify novel prognostic biomarkers related to BC progression utilizing weighted gene co-expression network analysis (WGCNA) and further bioinformatic analysis. First, we constructed a co-expression network by using WGCNA among 274 TCGA-BLCA patients and preliminarily screened out four genes (CORO1C, TMPRSS4, PIK3C2B, and ZNF692) associated with advanced clinical traits. In support, GSE19915 and specimens from 124 patients were used to validate the genes selected by WGCNA; then, CORO1C and TMPRSS4 were confirmed as hub genes with strong prognostic values in BC. Moreover, the result of gene set enrichment analysis (GSEA) and gene set variation analysis (GSVA) indicated that CORO1C and TMPRSS4 might be involved in the process of epithelial to mesenchymal transition (EMT) reversely. In addition, high expression of CORO1C was found to be significantly correlated with tumor-infiltrating neutrophils (TINs), a negative regulatory component that facilitates tumor distant progression and induces poor clinical outcome. In conclusion, our study first identified CORO1C and TMPRSS4 as vital regulators in the process of tumor progression through influencing EMT and could be developed to effective prognostic and therapeutic targets in future BC treatment.

## Introduction

Bladder cancer is the 10th most common cancer in the world with an estimated 549,000 new cases and 200,000 deaths in 2018 (1). The median age of tumor diagnosis is 73 years old, which is the most elderly of diagnosis among all cancer sites (2), and the mortality rate increased by 1% per year from 2000 to 2016 in the oldest old men (3), which means the probability of most bladder cancer cases occurring with tumor progression and advanced disease before diagnosis still ranks high. Current recognized treatments are radical resection and transurethral resection of bladder tumor (TURBT) for muscle invasive bladder cancer (MIBC) and those treatments with or without intravesical therapy for non-muscle invasive bladder cancer (NMIBC). However, ~60% of NMIBC patients will undergo recurrence (4, 5) following the surgery due to rapid tumor invasive growth and high tendency of distant progression. Therefore, for such difficulties in early tumor diagnosis and dealing with the frequent recurrence rate, there remains an urgent need to explore novel effective prognostic biomarkers and potential therapeutic targets in bladder cancer.

The majority of researchers have just focused on the differential expression of genes associated with BC and have largely ignored the high degree of interconnectivity among genes, where genes with semblable expression patterns might be functionally correlated. Weighted gene co-expression network analysis (WGCNA) is a distinct approach in identifying modules with different categories of correlated genes, and candidate biomarkers or therapeutic targets for cancer management can be picked out according to the connectivity between the internal linkage of the gene sets in specific modules and by calculating the association between the gene sets and related clinical features (6–9). Furthermore, WGCNA has been successfully applied to identify biomarkers or potential therapeutic targets for BC (8, 10). Hence, we attempt to construct a network through a comprehensive bioinformatics method in the light of WGCNA and identified prognostic genes of BC.

In this study, we first extracted the level-three mRNA expression data and clinical information of 274 bladder cancer patients from The Cancer Genome Atlas (TCGA) database for subsequent weighted network construction. By WGCNA, four modules and their candidate hub genes were preliminarily identified through evaluating the significance between modules and clinical traits. Survival analysis of the candidate hub genes was then performed in three data sets (TCGA-BLCA, GSE19915 data set, and clinical specimens), and CORO1C and TMPRSS4 were recognized as hub genes due to the consistency of prognostic value in overall survival (OS). The diverse gene set analysis method was later used to depict the potential biological functions of the hub gene. Finally, protein–protein interaction (PPI) network analysis was performed to investigate the relationships between the hub genes and the associated genes' network in corresponding modules.

## Results

### Co-expression Network Construction and Module Identification

**Figure 1:**
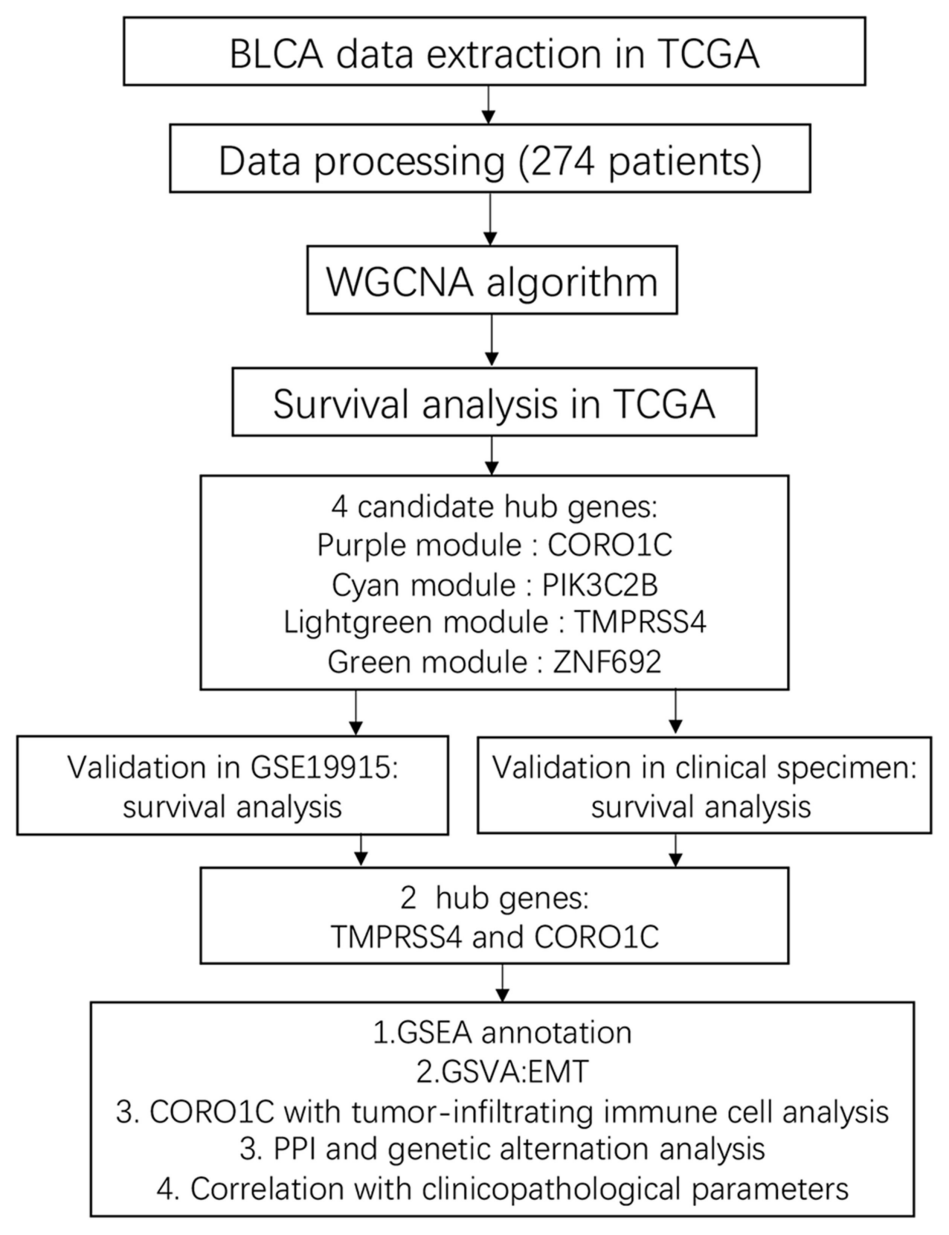
Flowchart design of the study.

**Figure 2:**
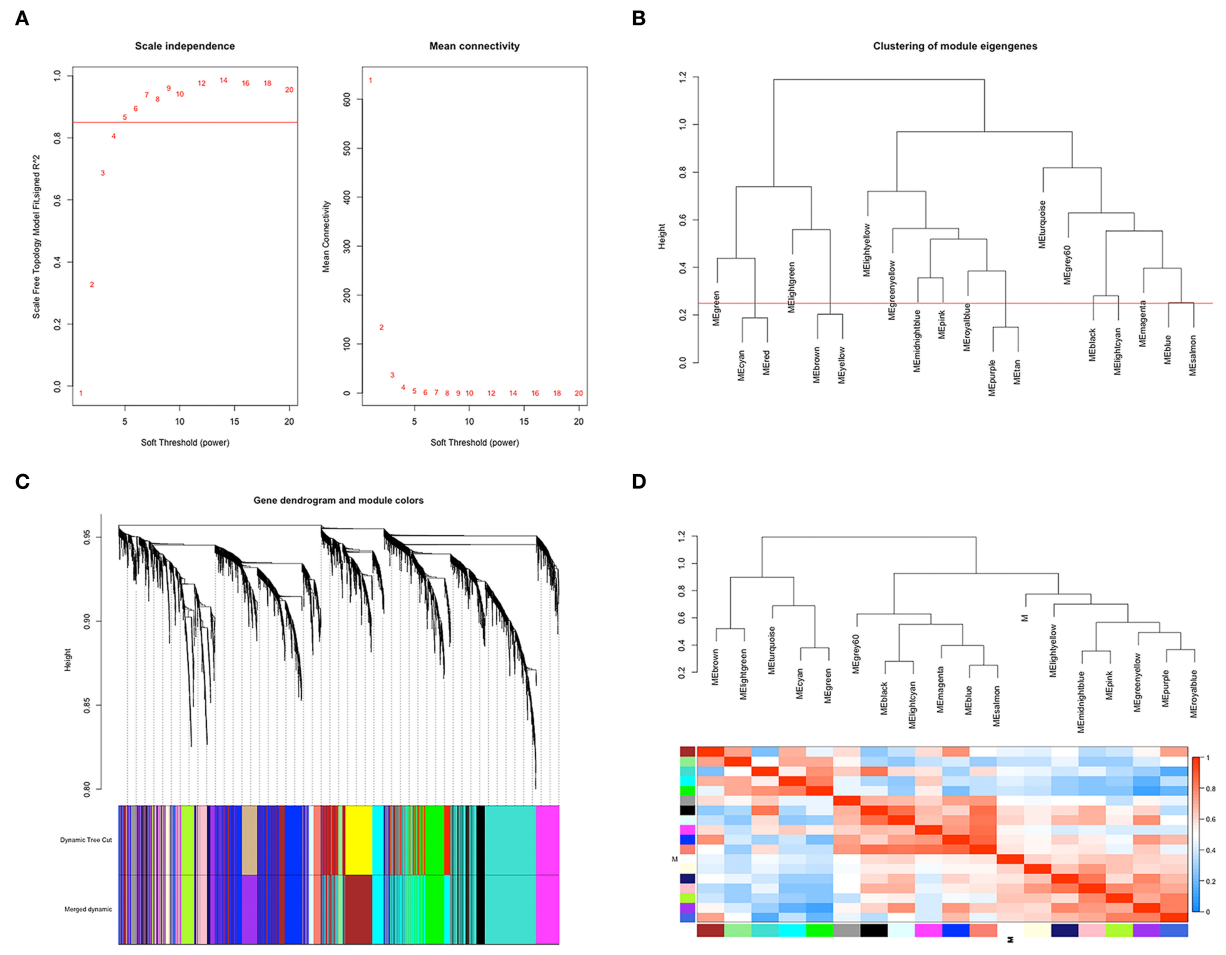
Co-expression network construction based on TCGA-BLCA mRNA data. **(A)** The analysis of topology for soft threshold powers and β = 5 (scale-free *R*^2^ = 0.85) was set as the soft thresholding for further adjacency calculation. **(B)** Clustering dendrograms were cut at a height of 0.25 to identify the similar modules. **(C)** The original 20 modules and merged 17 modules were displayed at top and bottom under the clustering dendrogram. **(D)** An eigengene network heat map showed the correlation between each module, and modules with close connectivity were clustered together.

According to the flowchart shown in Figure 1, after data processing, a total of 274 TCGA-BLCA samples and their selected clinical information were imported to WGCNA. In the present study, β = 5 (scale-free *R*^2^ = 0.85) was chosen as the soft thresholding for further adjacency calculation (Figure 2A). Genes in the gray module were regarded as non-specific genes and removed from further data processing. After merging the similar modules by a clustering height cutoff of 0.25 (Figure 2B), 17 modules possessing high credibility were finally generated in total with the initial modules and merged modules displaying under the clustering tree (Figure 2C). The candidate hub gene and module size of all 17 modules calculated by the “chooseTopHubInEachModule” function are shown in Table 1. According to the network adjacency of expression data, some modules indicated high levels of similarity, and three major clusters were formed by the “average” clustering method (Figure 2D). The heat map that demonstrates the relationship between seven clinical traits and each module identified by WGCNA is shown in Figure 2A. The cyan, turquoise, light green, and green modules were significantly negatively correlated not only with the M stage, but also with the papillary subtypes, grade, and T stage of bladder cancer. Interestingly, these three modules share high similarity between each other (Figure 2D), which implies that genes in such modules might work together to be protective regulators during tumor progression. Other modules, except the brown and grey60 modules, reflect significant positive correlation with most of the clinical traits, indicating that genes in these modules are more likely to promote advanced disease.

**Table 1:** The candidate hub gene and gene numbers of all 17 modules determined by WGCNA.

**Module colors**	**Gene frequency**	**Candidate hub gene**
Black	283	NUDC
Turquoise	935	NDUF83
Lightyellow	39	PIM2
Grey60	59	C1QBP
Pink	270	SELPLG
Purple	395	CORO1C
Blue	793	ECT2
Salmon	144	PPM1G
Brown	671	CNOT1
Cyan	422	PIK3C2B
Light green	45	TMPRSS4
Green yellow	170	EMILIN1
Royal blue	31	ITGAV
Magenta	254	ILF2
Midnight blue	115	UBE2L6
Green	291	ZNF692
Light cyan	83	NELFB

### Selection of Candidate Hub Genes From Identified Modules

**Figure 3:**
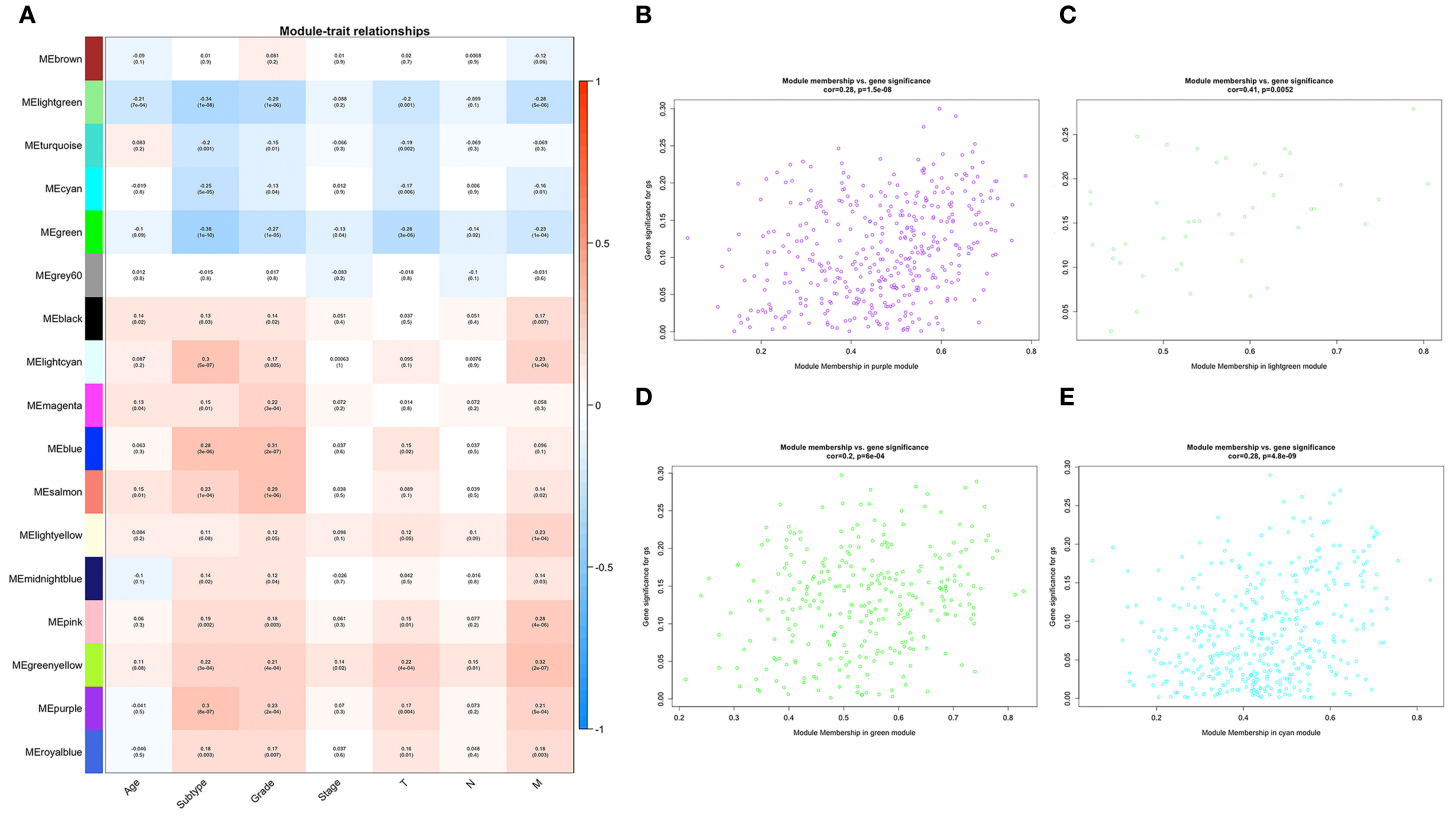
Relationships between WGCNA identified modules and clinical traits. **(A)** Heat map of the correlation among all 17 modules and seven selected clinical traits including age, tumor subtype (papillary and non-papillary), tumor grade (low grade and high grade), tumor stage (stage II and stage III-IV), tumor T stage (T2 and T3-4), tumor N stage (non-metastasis and metastasis), and tumor M stage (non-metastasis and metastasis). **(B)** Scatterplot of the relationship between gene significance (GS) and module membership (MM) in the purple module, **(C)** in the light green module, **(D)** in the green module, and **(E)** in the cyan module.

**Figure 4:**
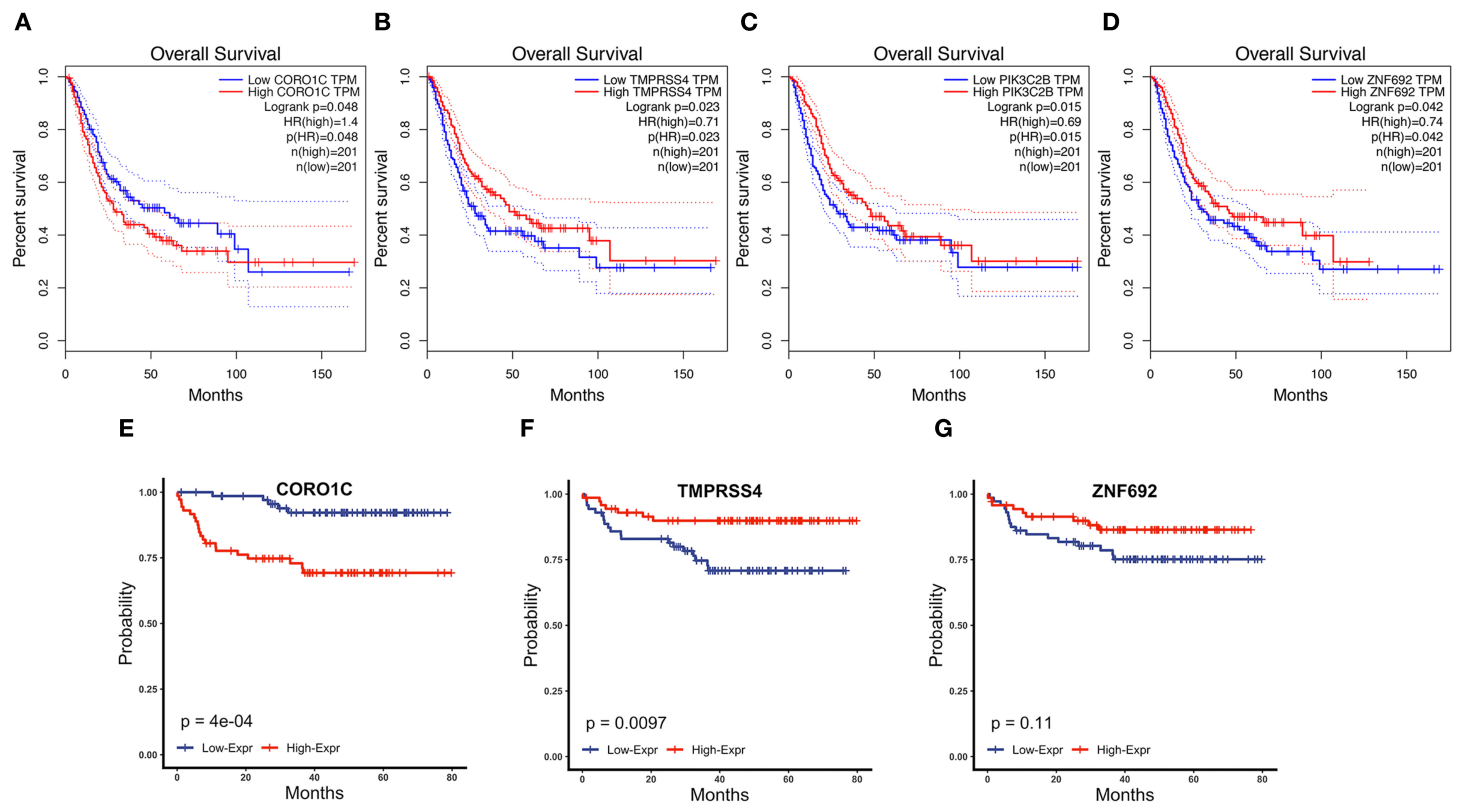
Survival analysis of four candidate hub genes in TCGA-BLCA and GSE19915 data. **(A–D)** Survival analysis result of CORO1C **(A)**, TMPRSS4 **(B)**, PIK3C2B **(C)**, and ZNF692 **(D)** in TCGA-BLCA downloaded from GEPIA. **(E–G)** Survival analysis of CORO1C **(E)**, TMPRSS4 **(F)**, and ZNF692 **(G)** in GSE19915.

To determine the prognostic values of the candidate hub genes in modules found by WGCNA, which as least significantly associated with one clinical trait (Figure 3A), the GEPIA online tool was used to perform survival analysis among them in TCGA-BLCA patients, and finally, four of all 15 candidate hub genes were identified to be valuable predictors of overall survival. Only the significant results were presented in Figures 4A–D. These four candidate hub genes (CORO1C, TMPRSS4, PIK3C2B, and ZNF692) were retained for further validation. Then, we selected the corresponding purple, light green, cyan, and green modules in order to evaluate their relationship with the M stage because metastasis was the primary cause of bladder cancer progression and treatment failure. As shown in Figures 3B–E, the relationship between gene significance (GS) and module membership (MM) are plotted to demonstrate that characteristic genes in these four modules are significant contributors to tumor M stage, and hub genes representing light green, cyan, and green modules could reverse tumor progression, but in the purple module, it could promote tumor progression (Figure 3A).

### Validation of the Candidate Hub Genes by Immunohistochemistry and GSE19915

**Figure 5:**
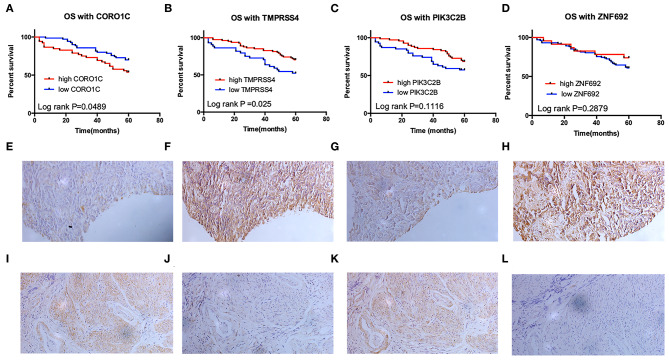
Validation of the prognostic value of four candidate hub genes in clinical specimens. **(A–D)** Survival analysis of CORO1C **(A)**, TMPRSS4 **(B)**, PIK3C2B **(C)**, and ZNF692 **(D)** in 124 bladder cancer patients' follow-up in our center. **(E–L)** Representative image of 200× immunohistochemical experiment on CORO1C **(E,F)**, TMPRSS4 **(G,H)**, PIK3C2B **(I,J)**, and ZNF692 **(K,L)** clinical specimens.

Clinical specimens for immunohistochemical analysis were collected from a cohort of 124 bladder cancer patients to clarify the expression pattern of the candidate hub genes (CORO1C, TMPRSS4, PIK3C2B, and ZNF692) identified in the above modules, and the main findings were shown in Figures 5E–L. Immunohistochemical staining revealed that, compared with other subcellular localization, these candidate hub genes were more highly expressed in the cytoplasm of tumor cells: 58.06% cases of bladder cancer specimens (72/124) were scored as CORO1C overexpression (Figures 5E,F), 34.68% cases of bladder cancer specimens (43/124) were scored as TMPRSS4 overexpression (Figures 5G,H), and 43.55% (54/124) and 81.45% (101/124) were scored as PIK3C2B and ZNF692 overexpression. To explore whether the candidate hub genes play consistent prognostic roles in clinical outcomes, overall survival analyses were performed on this immunohistochemical cohort. In particular, CORO1C and TMPRSS4 were noted significant (Figures 5A–D). We downloaded another online data set, GSE19915, which included 144 bladder cancer patients to test the prognostic performance of these genes, but only three genes (CORO1C, TMPRSS4, and ZNF692) could match the probes in later survival analysis (Figures 4E–G). We also assessed the association between candidate hub genes and related clinicopathological features. The relationship between the expression level of hub genes and clinicopathological parameters, including age, gender, stage, TNM stages, and grade, were concluded in Tables 2A,B. Notably, among all 274 TCGA-BLCA patients, CORO1C were significantly differentially expressed between the groups of T stage category, M stage category, and grade. It's evident that CORO1C overexpression was highly associated with advanced disease and worse clinical status in bladder cancer. Contrary to CORO1C, the expression level of TMPRSS4 was significantly higher in the groups of young age (<60 years old), early T stage, well-differentiated tumor patients, and cases without lymph node or distant metastasis, which provided strong evidence of the role that TMPRSS4 played in better clinical outcome involvement. The expression of CORO1C and TMPRSS4 in normal bladder tissues and the relationship between two genes and tumor stage were explored via GEPIA online tool (Supplementary Figure 1). The results show that the higher TMPRSS4 expression indicates the lower tumor stage.

**Table 2A:** The correlations between the CORO1C and clinicopathological features based on TCGA-BLCA data.

**Clinicopathological parameters**	***n***	**Mean ±*SD***	***p*-value**
Age (years)			0.1138
<60	70	16.7603 ± 12.4954	
≥60	204	17.9346 ± 10.4981	
Gender			0.4653
Male	202	17.3079 ± 10.8785	
Female	72	18.5508 ± 11.4752	
Stage			0.9494
Stage II–III	180	17.8167 ± 11.2904	
Stage IV	94	17.2857 ± 10.5661	
T category			0.0042^*^
T2	95	15.1989 ± 9.8401	
T3–T4	179	18.9772 ± 11.4297	
N catagory			0.9832
No	183	17.7768 ± 11.2386	
Yes	91	17.3486 ± 10.6557	
M catagory			0.0002^*^
No	129	15.3059 ± 10.3289	
Yes	145	19.7062 ± 11.2547	
Grade			0.0002^*^
High grade	257	18.1919 ± 11.0845	
Low grade	17	9.2089 ± 5.4622	

* *Represented statistical significance p < 0.01*.

**Table 2B:** The correlations between the TMPRSS4 and clinicopathological features based on TCGA-BLCA data.

**Clinicopathological parameters**	***n***	**Mean ±*SD***	***p*-value**
Age (years)			0.0026^*^
<60	70	30.1060 ± 29.5519	
≥60	204	19.7486 ± 28.5467	
Gender			0.9064
Male	202	23.6351 ± 31.065	
Female	72	18.9148 ± 22.5550	
Stage			0.0039^*^
Stage II–III	180	25.3212 ± 30.0975	
Stage IV	94	16.7907 ± 26.3627	
T category			0.0001^*^
T2	95	28.7322 ± 31.8749	
T3-T4	179	19.0312 ± 20.0176	
N catagory			0.0033^*^
No	183	25.5201 ± 30.5025	
Yes	91	16.1095 ± 25.0689	
M catagory			0.0002^*^
No	129	28.4354 ± 32.4548	
Yes	145	17.0205 ± 24.6583	
Grade			<0.0001^*^
High grade	257	20.1151 ± 27.2240	
Low grade	17	56.8559 ± 35.3721	

* *Represented statistical significance p < 0.01*.

In general, good consistency of CORO1C and TMPRSS4's prognostic values for overall survival outcome has been observed among three data sets, robustly indicating that CORO1C is a risk factor in bladder cancer development while TMPRSS4 is a protective factor, and as hub genes, their relationships with clinicopathological features are in accord with the purple and light green modular characterizations that have been described by WGCNA analysis (Figure 3A).

### Pathway Analysis of the Hub Genes

**Figure 6:**
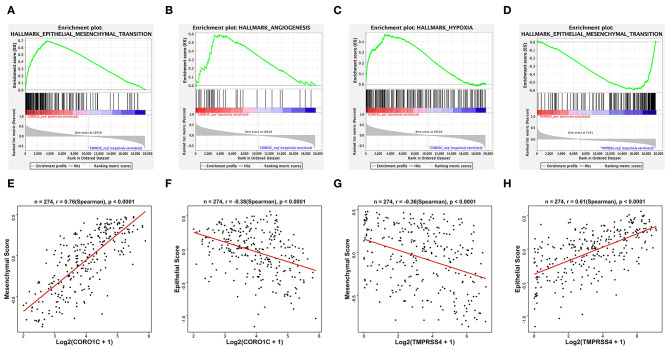
Hallmark GSEA and EMT GSVA of hub genes based on TCGA-BLCA mRNA data. Some representative top enriched pathways of CORO1C were **(A)** epithelial to mesenchymal transition pathway, **(B)** angiogenesis pathway, and **(C)** hypoxia pathway. Only one downregulated GSEA result of TMPRSS4 enriched in **(D)** mesenchymal transition pathway. **(E)** GSVA pathway score of mesenchymal state gene set and **(F)** epithelial state gene set vs. normalized Log2(FPKM + 1) expression of CORO1C. **(G)** GSVA pathway score of mesenchymal state gene set and **(H)** epithelial state gene set vs. normalized Log2(FPKM + 1) expression of TMPRSS4.

To investigate the potential molecular functions of CORO1C and TMPRSS4 in BC, we performed GSEA and GSVA based on the processed TCGA-BLCA data. The GSEA results of hallmark gene sets showed genes that have a positive correlation with CORO1C are significantly enriched in the following pathways: epithelial to mesenchymal transition pathway, angiogenesis pathway, and hypoxia pathway (Figures 6A–C). Both the angiogenesis and hypoxia pathways are closely related to the occurrence and development of EMT-associated tumor metastasis. Accumulating research has reported that a hypoxic tumor microenvironment is a key regulator in affecting tumor EMT transformation and active angiogenesis would facilitate distant transportation of EMT-progressing tumor cells through the microvasculature while abnormal vasculatures in the tumor microenvironment also led to a hypoxic condition (11–13). Conversely, TMPRSS4-related downregulated genes were only significantly enriched in the epithelial to mesenchymal transition pathway (Figure 6D) while upregulated genes did not observe enrichment in any pathways. To further verify the relationship between EMT and hub genes, the GSVA method was utilized to evaluate the EMT pathway score variation. The Spearman correlation coefficient shows a significantly strong positive correlation (*r* = 0.76, *p* < 0.0001) between CORO1C expression and mesenchymal pathway (Figures 6E,F), and the correlation between CORO1C and epithelial pathway is significantly negative (*r* = −0.38, *p* < 0.0001). On the contrary, TMPRSS4 has a significantly strong positive correlation with genes in epithelial pathways (*r* = 0.61, *p* < 0.0001) but is opposite to the mesenchymal process in bladder cancer (*r* = −0.36, *p* < 0.0001) (Figures 6G,H). In summary, this result elucidates that there is a high possibility of the participation of CORO1C and TMPRSS4 in the bladder tumor cell EMT based on the opposite effects, which suggested their different roles during disease metastatic course.

### CORO1C Expression and TME Evaluation

**Figure 7:**
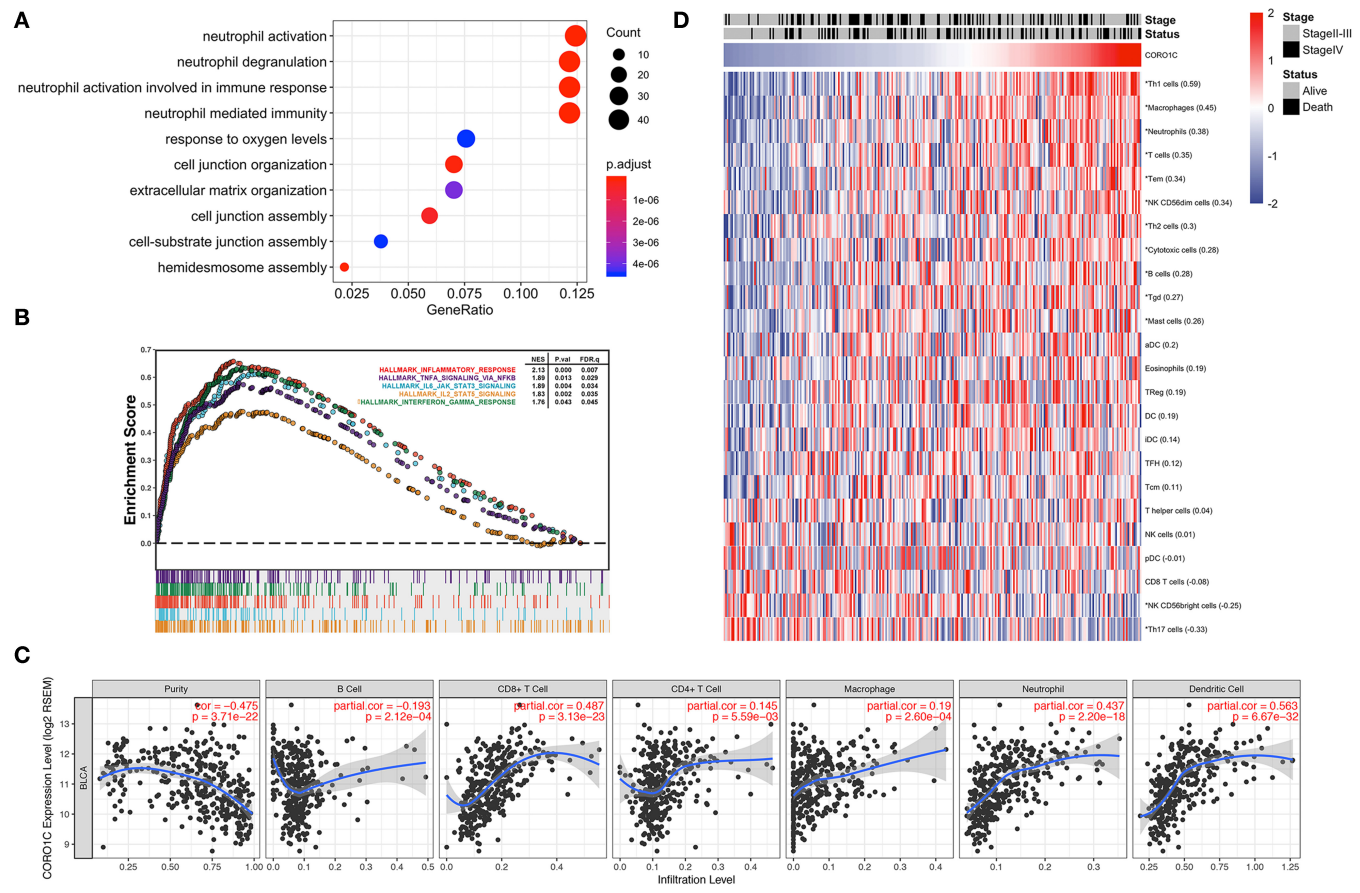
Evaluation of CORO1C expression and tumor immune infiltration based on TCGA-BLCA data. **(A)** Biological process of gene ontology enrichment for WGCNA purple module. **(B)** Integrated plot presented some top GSEA enriched immune-related pathway associated with CORO1C expression. **(C)** Evaluation of CORO1C expression level and immune infiltration level in BC via TIMER tool. **(D)** Relationship between CORO1C and 23 types of immune cells via ssGSEA analysis. The Pearson correlative coefficients were marked following the name of each immune components (“*” represented statistical significance *p* < 0.01).

To better demonstrate the accordance between hub genes and corresponding modules, GO_BP enrichment analysis was performed in the purple and light green modules by R package “clusterProfiler.” Ontology analysis of the light green module did not find any significantly enriched pathways, and genes in the purple module were positively related to the top four enriched processes of neutrophil activation, neutrophil degranulation, neutrophil activation involved in the immune response, and neutrophil-mediated immunity (Figure 7A). The hallmark GSEA results of the hub gene CORO1C indicate that many pathways associated with the immune process were significantly enriched, such as interferon gamma response, inflammatory response, IL6-JAK-STAT3 signaling, IL2-STAT5 signaling, and TNFα signaling via NF-κB (Figure 7B). In addition, the TIMER online tool was exerted to evaluate the potential relationships between the expression of CORO1C and both tumor purity score as well as six types of tumor-infiltrating immune cells (Figure 7C). Based on the linear least square regression calculating method, the expression of CORO1C was shown to have a negative tendency with tumor purity (*r* = −0.475, *p* = 3.71e-22) and the level of infiltrating B cell (*r* = −0.193, *p* = 2.12e-04); meanwhile, CORO1C expression was positively correlated with the infiltrating level of CD8^+^ T cells (*r* = 0.487, *p* = 3.13e-23), CD4^+^ T cells (*r* = 0.145, *p* = 5.59e-03), macrophages (*r* = 0.19, *p* = 2.60e-04), neutrophils (*r* = 0.437, *p* = 2.20e-18), and dendritic cells (*r* = 0.563, *p* = 6.67e-32). The ssGSEA method was selected as another estimation tool to verify the probable relationship between CORO1C expression and 23 types of immune components with a heat map displaying the order ranked from high to low correlation appraised by Pearson correlation (Figure 7D). Interestingly, the relation between CORO1C and neutrophil share the strongest consistency among different means, which also confirms the hub role of CORO1C in a neutrophil mainly related immune infiltrating module.

### PPI and Genetic Alteration Characterizations

**Figure 8:**
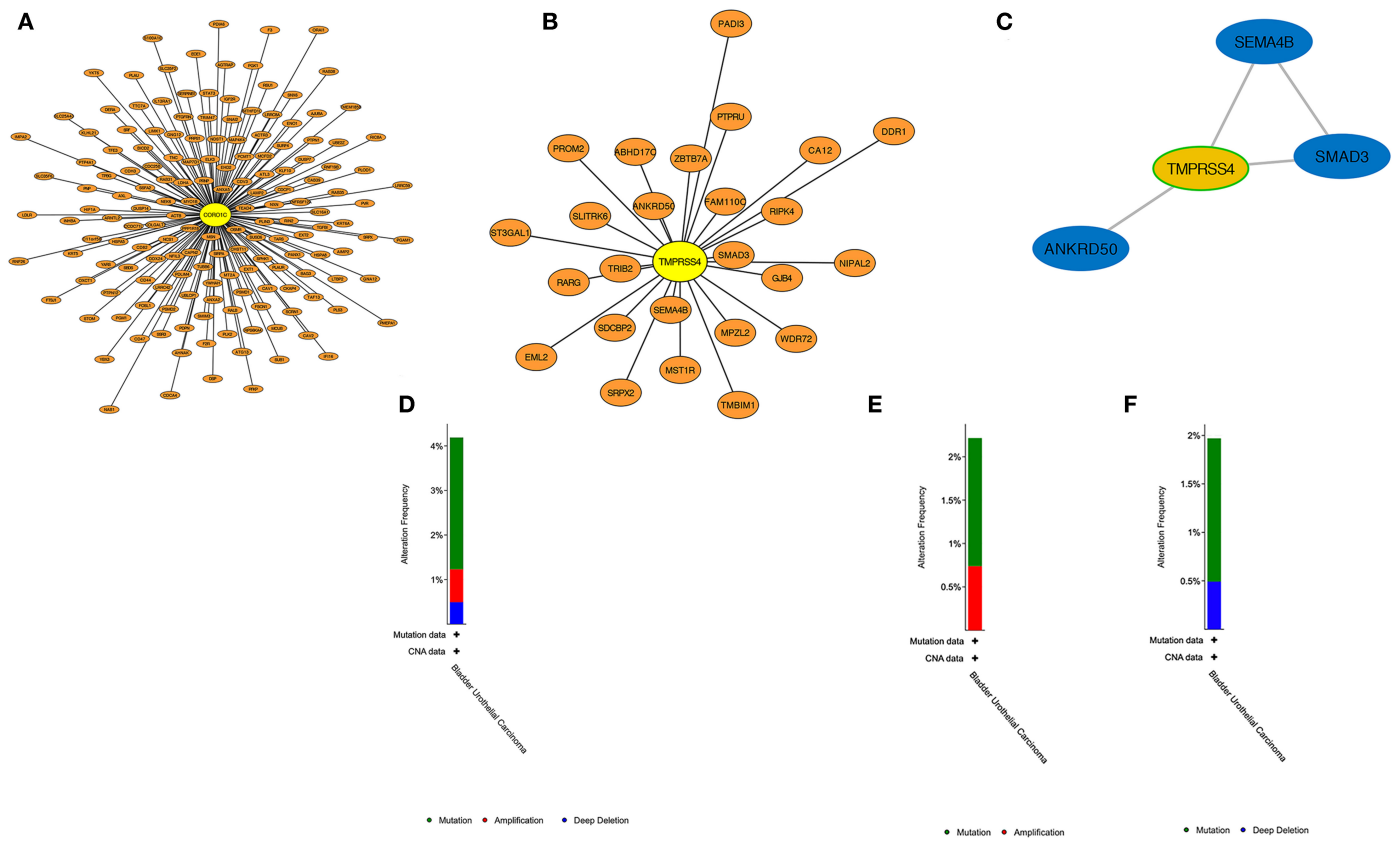
PPI construction and genetic alteration analysis. **(A)** In WGCNA purple modules, genes highly related to CORO1C were established in a protein network. **(B)** In WGCNA light green modules, protein interaction of genes highly related to TMPRSS4. **(C)** The strongest weighted genes network in WGCNA light green module. **(D)** The overall genetic alternation status of two hub genes in TCGA-BLCA data. **(E)** The alternation type and frequency of CORO1C and **(F)** TMPRSS4, respectively, in TCGA-BLCA data.

PPI construction was performed with the aim to explore the interaction network between hub genes and other genes in the corresponding modules. As shown in Figures 8A,B, 163 genes were obtained from the WGCNA purple module out of 394 genes, and 25 genes were obtained in the WGCNA light green module out of 44 genes that were generated by taking the intersection with the highly correlated genes identified from hub genes' Pearson analysis (*r* > 0.4). It means that these screened-out genes would form crosslink networks with the hub genes and could well represent the major functional modules. Because the number of genes in the light green module is rather small, the connectivity between TMPRSS4 and other genes in this module were evaluated by setting up the adjacency threshold as 0.02, and finally, a weighted network with highest strengths containing only four genes was structured to represent the module (Figure 8C). The total alteration frequency is included to illustrate that CORO1C and TMPRSS4 are altered in 4.2% cases, which looks relatively not much higher in bladder cancer (Figure 8D). We then separately assessed the gene alternation type and mutational rates of *CORO1C* and *TMPRSS4* in TCGA-BLCA data. The two genes had almost identical mutational rates, both around 2%, but the major genetic changes in *CORO1C* are mutation and copy number amplification while deep depletion of *TMPRSS4* gene is detectable in a small group of patients, which suggests a heterogeneous gene alteration pattern and exclusive molecular function of these two genes (Figures 8E,F).

## Discussion

Bladder cancer is the most common urinary malignancy with the characteristic of high incidence and mortality rate. Currently, radical cystectomy is effectively exerted on patients at an early stage, but in many cases, patients suffer tumor recurrence within 5 years (4, 14, 15), and those who are diagnosed with advanced disease could barely benefit from such therapeutic approaches, which suggests recurrence management and early diagnosis remain the major challenges in bladder cancer improvement. Therefore, there is an urgent need to find new biomarkers that accurately predict the tumor progressive status and clinical prognosis of bladder cancer.

EMT is a cellular process that is known to be essential for malignant progression, which could give tumor cells potential capability in further local invasion and distant metastasis. Moreover, cancer cells with stromal features are more resistant to traditional therapy, and this is the main reason accounting for death and treatment failure (16, 17). In the process of driving tumor progression, EMT has been proven to be a set of multiple dynamic transition states between the epithelial and mesenchymal phenotypes rather than a process involving a single binary decision, and different EMT stages have been identified in multiple tumor subpopulations: epithelial states, intermediate hybrid states, and completely mesenchymal states (18, 19). In bladder urothelial carcinoma, tumor metastasis caused by EMT is the main cause of death, and reversing EMT has been widely considered to be a method to combat the progression of bladder cancer in treatment (20, 21). The reprogramming of gene expression during EMT, as well as non-transcriptional changes, is initiated and controlled by signaling pathways that respond to extracellular cues (22). Interestingly, we use GSEA and GSVA to explore the function of TMPRSS4 and CORO1C, and we find that both TMPRSS4 and CORO1C are significantly enriched in pathways related to epithelial–mesenchymal transition but with a completely adverse direction. CORO1C was positively correlated with the process of EMT, and TMPRS4 is negatively correlated with the process of EMT. The correlation analysis between EMT gene sets and the hub genes also supports the discovery from GSEA. Therefore, we speculate that these two genes may play key roles in the pathogenesis of bladder cancer via influencing EMT.

Transmembrane protease serine 4 (TMPRSS4), a novel type II transmembrane serine protease, has been proven to have a crucial role in multiple tumors. In lung squamous cell carcinoma, overexpressed TMPRSS4 is associated with disease recurrence and poor survival by promoting invasion and metastasis (23–25). TMPRSS4 is overexpressed in breast cancer cells, and its overexpression promoted the proliferation, migration, and invasion of breast cancer cells and is related to poor prognosis (26). In colorectal cancer, the high expression state of TMPRSS4 is significantly correlated with advanced TNM stage and predicted poor prognosis (27). In thyroid cancer, TMPRSS4 is detected to be significantly associated with the promotion of proliferation in thyroid cancer cells (28). In gastric cancer, upregulation of TMPRSS4 facilitates cell migration and invasion through the activation of NF-κB/MMP-9 signaling (29). In pancreatic carcinoma, TMPRSS4 is identified as a candidate biomarker by affecting the clonability and invasiveness of pancreatic cancer cells, and overexpressed TMPRSS4 predicts poor prognosis (30, 31). In prostate cancer cells, overexpressed TMPRSS4 promotes migration via the progression of EMT (32). Interestingly, in contrast to most cancer types in published research, our study finds that high expression of TMPRSS4 predicts a better clinical outcome in bladder cancer, and this finding has been verified in three data sets (two online and one from our center). The relationship between TMPRSS4 expression level and clinical traits also supports the protective role of TMPRSS4 in bladder cancer, which might be related to its counteractive function in the EMT process.

Coronin-like actin binding protein 1C (CORO1C), a member of the WD repeat protein family, regulates the actin-dependent process by assembling F-actin. In triple-negative breast cancer (TNBC) cells, miR-206 inhibits cancer cell migration by directly targeting CORO1C, which regulates actin filaments (33). In breast cancer MDA-MB-231 cells, YBX1 could regulate invasion and migration by regulating its downstream target CORO1C (34). In lung squamous cell carcinoma, CORO1C was identified as a target of the miR-1/133a cluster, and silencing CORO1C inhibited cancer cell proliferation, migration, and invasion (35). In gastric cancer, overexpressed CORO1C is associated with poor prognosis and could promote metastasis by regulating cyclin D1 and vimentin (22). In our study, the high expression level of CORO1C also predicted poor survival outcomes in bladder cancer, which is consistent with other cancer types.

The tumor microenvironment (TME) is the supportive nest for tumor cells and can be divided into two parts: tumor cells and the surrounding matrix. The matrix consists of a variety of cells, including immune cells, microvascular cells, lymphoid endothelial cells and fibroblasts, soluble factors, signaling molecules, and extracellular matrix (36). During tumor development, a series of complex changes occurs in the tumor microenvironment, including the immune response and infiltration of various immune cells, especially neutrophils, which affect the initiation and development of tumors (37). Many studies have shown that TINs promote the adhesion of tumor cells to the epithelial monolayer and, thus, accelerate the metastasis of tumor cells (38, 39). In pancreatic ductal adenocarcinoma, TINs promote the EMT process and the invasive growth of tumor cells (40). In epithelial-derived tumors, an increasing number of neutrophils is usually a marker of the systemic inflammatory response that triggers the proliferation and metastasis of tumor cells by inhibiting apoptosis, motivating angiogenesis, and inducing DNA damage (41). Furthermore, neutrophils induce gastric cancer cells to occur EMT, leading to migration and invasion of tumor cells in TME (42). In nasopharyngeal carcinoma, CORO1C promotes cancer cell migration and invasion by induction of EMT (43). We infer that CORO1C may induce EMT in bladder cancer cells by increasing neutrophil activity. Our research first demonstrates that CORO1C positively relates to neutrophil activation, neutrophil degranulation, neutrophil activation involved in the immune response, neutrophil-mediated immunity, and EMT. These results support the view that CORO1C is highly positively correlated with EMT and ultimately leads to poor prognosis in bladder cancer patients.

In the present study, WGCNA is used to construct a co-expression network based on processed TCGA-BLCA data. We also assess clinical features and attempt to find key modules that are most relevant to the advanced stage of bladder cancer. Finally, four modules and their corresponding candidate hub genes are preliminarily identified for further validation. Survival analysis of these candidate hub genes is performed in three data sets (TCGA-BLCA, GSE19915, and an independent cohort of bladder cancer patients from our center). Finally, CORO1C and TMPRSS4 are screened out as hub genes that are significantly correlated with OS. CORO1C and TMPRSS4 might be two meaningful clinical indicators in bladder cancer but appear to have opposite prognostic value.

There are also some limitations in this study. First, the molecular function of CORO1C and TMPRSS4 in EMT need to be verified by biological experiments *in vitro*, which is in our future research plan. Second, the concrete relationship between CORO1C and neutrophils should be tested by co-culture or by immunohistochemical marker staining. Overall, through the integrated analysis of multiple data sets and the establishment of a co-expression network by WGCNA, CORO1C and TMPRSS4 are found participating in the tumor progression with valuable prognostic information in predicting bladder cancer patients' outcome and might be appealing treatment targets in clinical decision making.

## Materials and Methods

### Bladder Cancer Data Sources and Processing

The level three RNA-seq data and corresponding clinical information of 412 BC patients were downloaded from the TCGA database (https://cancergenome.nih.gov/). The exclusion criteria were described as follows: (1) neoadjuvant chemotherapy history, (2) unclear histological type of tumor, (3) T0 or T1 stage of tumor, (4) inadequate follow-up information (missing or <1 month), and (5) grade version not belonging to the American Joint Committee on Cancer (AJCC) 6th or 7th edition. Eventually, 274 patients were included in this study to perform WGCNA. The microarray data set GSE19915 consists of 144 BC patients, and related clinical data were downloaded from the Gene Expression Omnibus database (https://www.ncbi.nlm.nih.gov/geo/) to verify the candidate hub genes found by WGCNA.

### Construction of a Weighted Gene Co-expression Network and Identification of Candidate Hub Genes

A matrix includes the expression data of top 5,000 genes ranked by median absolute deviation (MAD), which, retrieved from 274 TCGA-BLCA samples, was used to construct a weighted gene co-expression network by R package “WGCNA” (6). First, to construct a reliable sample tree, the samples with connectivity less than −2.5 were filtered as outliers. Second, multiple soft thresholding powers ranging from 1 to 20 were established to select the appropriate indices (*R*^2^ = 0.85) for a scale-free topology network construction with enhanced strong Pearson correlations and weakened weak correlations between paired genes in the adjacency matrix. The adjacency matrix was then converted into a topological overlap matrix (TOM), which could evaluate the network connectivity of a gene, and the corresponding dissimilarity (dissTOM) was generated by subtracting TOM from 1. According to the TOM-based dissimilarity measure, genes were divided into different gene modules through the dynamic tree cut method with a minimum module group size of 30 for the resulting average linkage hierarchical clustering dendrogram, and the modules with low credibility were identified and merged together with a height cutoff of 0.25 in the clustering. The similarity of each module was evaluated by the adjacency of the expression data and the similar modules were clustered together. Later, seven BLCA clinical traits (including patient age, histological subtype of papillary and non-papillary tumors, histological grade, tumor stage, and TNM information) were selected, and the Pearson correlation with co-expression modules was calculated and visualized by a heat map. Finally, the “chooseTopHubInEachModule” function embedded in the “WGCNA” package was used to recognize the candidate hub genes that have the highest connectivity in each module.

### Survival Analysis of the Candidate Hub Genes

Prognostic values of the candidate hub genes in TCGA were assessed by Gene expression profiling interactive analysis (GEPIA, http://gepia.cancer-pku.cn) online tools. Survival analysis in the GSE19915 data set was conducted for the candidate hub genes using “survival” R package (https://CRAN.R-project.org/package=survival). Tumor samples within the above data sets were divided based on the candidate hub gene's median expressive value, and the survival analysis was estimated by the Kaplan–Meier (K-M) survival curves of each patient with overall survival (OS) as the end point.

### Functional Annotation of the Hub Genes

GSEA was carried out using the Java GSEA v4.0.1 desktop program (http://software.broadinstitute.org/gsea/datasets.jsp). Fifty referenced Hallmark gene sets from MSigDB (http://software.broadinstitute.org/gsea/msigdb/index.jsp) were downloaded to identify significant pathways associated with hub gene expression. Gene ontology (GO) biological process annotation of the hub gene associated modules was implemented by R package “clusterProfiler” (44). In the gene set enrichment analysis, large sets with more than 500 genes and small sets with <15 genes were excluded from the results, and enriched pathways with a cutoff threshold of *p* < 0.05 and FDR < 0.25 were considered valuable.

### Variation Analysis Between the Hub Genes and EMT Gene Sets

Based on the integration of published literature, EPITHELIAL-MESENCHYMAL TRANSITION GENE DATABASE (dbEMT2, http://dbemt.bioinfo-minzhao.org/index.html), EMT-related gene sets, epithelial-related gene sets, and mesenchymal-related gene sets selected from MSigDB (http://software.broadinstitute.org/gsea/msigdb/index.jsp), finally 370 epithelial-related genes and 733 mesenchymal-related genes were collected and further processed into an epithelial-related gene set and a mesenchymal-related gene set, respectively. The enrichment scores of the epithelial-related gene set and mesenchymal-related gene set were calculated by the “GSVA” package (45). Spearman correlation between epithelial or mesenchymal gene set enrichment score and the hub gene Log2 transformed expression data were estimated to evaluate the relationship between the hub genes and the EMT process.

### Tumor-Infiltrating Immune Cell Analysis

Immunologic infiltration data were collected from the Tumor Immune Estimation Resource (TIMER, https://cistrome.shinyapps.io/timer/) platform to explore the correlation between hub genes and tumor-infiltrating immune components. TIMER is a comprehensive resource for the systematic analysis of immune infiltrates across diverse cancer types. The abundance of six immune cells (B cell, CD8^+^ T cell, CD4^+^ T cell, macrophage, neutrophil, and dendritic cell) were evaluated by statistical methods mining sequencing data retrieved from TCGA and validated using pathological estimations (46, 47). To calculate the consistency of results retrieved from TIMER, we collected 12 innate immune cells and 12 adaptive immune-related cytotoxic components with the marker genes previously identified by Bindea et al. (48) for further estimating by single sample GSEA (ssGSEA), an extension embedded in the “GSVA” R package. The quantification of RNA-seq abundance was first standardized by calculating the transcripts per million (TPM) to estimate the relative degree of the tumor-infiltrating immune microenvironment among 274 TCGA-BLCA tumor samples; after transforming the ssGSEA scores into a *z*-score distribution, the normalized enrichment score of each immune component was ranked from high to low by the Pearson correlation with the expression of CORO1C and displayed in a heat map form.

### Protein Network Construction and Genetic Alteration of the Hub Genes

We identified 1,273 genes that were highly correlated with the expression of CORO1C and 164 genes that were highly correlated with the expression of TMPRSS4, according to the strict criteria of Pearson *r* > 0.4 and *P* < 0.0001. Finally, 163 genes that were regarded as the most closely related genes with CORO1C were obtained from the intersection of 1,273 correlative gene sets and all 394 genes contained in the WGCNA purple module (excluding CORO1C). Another 25 genes most closely related to TMPRSS4 were also determined by the same method as described above. Cytoscape software version 3.5.0 (https://cytoscape.org) was downloaded to visualize the PPI network of the hub genes and their possible co-expressed relationship. The genetic alteration type and mutational frequency map of the hub genes were assessed through the cBioPortal for Cancer Genomics (https://www.cbioportal.org) (49).

### Patients and Follow-Up

The study was approved by the Ethics Committee of the First Affiliated Hospital of China Medical University with informed consent obtained from all patients. All 124 BC patients have suffered radical resection or TURBT in our hospital between 2010 and 2012. According to the AJCC stage, there were 88 cases of NMIBC and 56 cases of MIBC. The mean follow-up period of patients was 49 months, and no patients were lost to follow-up after surgery. OS was defined as the time between surgical resection and death or the last follow-up.

### Immunohistochemistry of Tissue Specimens

After fixation with 10% buffered formalin at room temperature for 24 h, the specimens were embedded in paraffin blocks, and the samples were sliced into 4-μm-thick sections. Then, the sections were deparaffinized using a graded ethanol series. Antigen retrieval was performed by a combination of pressure and heat in citrate buffer (pH 6.0) for 60 s. Subsequently, we incubated the sections with a CORO1C polyclonal antibody (diluted 1:200; PA5-21775; Thermo Fisher Scientific; USA), TMPRSS4 polyclonal antibody (diluted 1:200; ab188816; Abcam; USA), PIK3C2B polyclonal antibody (diluted 1:200; ab231122; Abcam; USA), and ZNF692 polyclonal antibody (diluted 1:200; PA5-63794; Thermo Fisher Scientific; USA) overnight at 4°C. We used the streptavidin-peroxidase method to detect antibodies, and phosphate-buffered saline (PBS) was used as a substitute for the primary antibody as the negative control. Positive controls were used to ensure technique validity. All the sections were performed independently by two experienced pathologists. When evaluating the staining intensity, the staining degrees were classified as 0 (negative or weak), 1 (moderate), or 2 (strong). The percentage of positive cells was determined, and a score of 1 to 3 was defined as follows: 1 (<50%), 2 (50–75%), and 3 (75–100%). The patients were divided into two groups based on the result of multiplying the two scores: high expression (>3) and low expression (≤3).

### Statistical Analysis

All statistical analyses were conducted using SPSS 24.0 (Chicago, IL, USA) and R 3.6.0 (https://www.r-project.org/). R and GraphPad Prism 6 (San Diego, CA, USA) were used to draw plots. The Wilcox test was applied to compare the expression of hub gene between different clinical groups. *P* < 0.05 was considered statistically significant.

## Data Availability Statement

Publicly available datasets were analyzed in this study, these can be found in The Cancer Genome Atlas (https://cancergenome.nih.gov/).

## Ethics Statement

The studies involving human participants were reviewed and approved by the Ethics Committee of the First Affiliated Hospital of China Medical University. The patients/participants provided their written informed consent to participate in this study.

## Author Contributions

ZL and TL conceived the study. JY, LY, NW, TH, and ZS participated in statistical analysis. CW performed the experiments. CW and YY wrote the manuscript. LY revised the manuscript. All authors contributed to the article and approved the submitted version.

## Conflict of Interest

The authors declare that the research was conducted in the absence of any commercial or financial relationships that could be construed as a potential conflict of interest.

## Supplementary Material

Supplementary Figure 1 **(A)** The expression of CORO1C and TMPRSS4 in normal bladder tissues and bladder cancer tissues in the TCGA and GTEx database. **(B)** The relationship between two hub genes and tumor stage via GEPIA online tool.

## References

[1] Bray F Ferlay J Soerjomataram I Siegel RL Torre LA Jemal A. Global cancer statistics 2018: GLOBOCAN estimates of incidence and mortality worldwide for 36 cancers in 185 countries. CA Cancer J Clin. (2018) 68:394–424. 10.3322/caac.21492 30207593

[2] Botteman MF Pashos CL Redaelli A Laskin B Hauser R. The health economics of bladder cancer: a comprehensive review of the published literature. Pharmacoeconomics. (2003) 21:1315–30. 10.1007/BF03262330 14750899

[3] DeSantis CE Miller KD Dale W Mohile SG Cohen HJ Leach CR. Cancer statistics for adults aged 85 years and older, 2019. CA Cancer J Clin. (2019) 69:452–67. 10.3322/caac.21577 31390062 PMC12103238

[4] Sylvester RJ van der Meijden AP Oosterlinck W Witjes JA Bouffioux C Denis L. Predicting recurrence and progression in individual patients with stage Ta T1 bladder cancer using EORTC risk tables: a combined analysis of 2596 patients from seven EORTC trials. Eur Urol. (2006) 49:466–5; discussion 475–467. 10.1016/j.eururo.2005.12.031 16442208

[5] Davaro F Schaefer J May A Raza J Siddiqui S Hamilton Z. Invasive non-urachal adenocarcinoma of the bladder: analysis of the National Cancer Database. World J Urol. (2019) 37:497–505. 10.1007/s00345-018-2411-7 30030660

[6] Langfelder P Horvath S. WGCNA: an R package for weighted correlation network analysis. BMC Bioinformatics. (2008) 9:559. 10.1186/1471-2105-9-559 19114008 PMC2631488

[7] Clarke C Madden SF Doolan P Aherne ST Joyce H O'Driscoll L. Correlating transcriptional networks to breast cancer survival: a large-scale coexpression analysis. Carcinogenesis. (2013) 34:2300–8. 10.1093/carcin/bgt208 23740839

[8] Yan X Guo ZX Liu XP Feng YJ Zhao YJ Liu TZ. Four novel biomarkers for bladder cancer identified by weighted gene coexpression network analysis. J Cell Physiol. (2019) 234:19073–87. 10.1002/jcp.28546 30927274

[9] Zhang C Li Z Hu J Qi F Li X Luo J. Identification of five long noncoding RNAs signature and risk score for prognosis of bladder urothelial carcinoma. J Cell Biochem. (2019) 121:856–66. 10.1002/jcb.29330 31373406

[10] Giulietti M Occhipinti G Righetti A Bracci M Conti A Ruzzo A. Emerging biomarkers in bladder cancer identified by network analysis of transcriptomic data. Front Oncol. (2018) 8:450. 10.3389/fonc.2018.00450 30370253 PMC6194189

[11] Song R Song H Liang Y Yin D Zhang H Zheng T. Reciprocal activation between ATPase inhibitory factor 1 and NF-kappaB drives hepatocellular carcinoma angiogenesis and metastasis. Hepatology. (2014) 60:1659–73. 10.1002/hep.27312 25042864

[12] Tsai YP Chen HF Chen SY Cheng WC Wang HW Shen ZJ. TET1 regulates hypoxia-induced epithelial-mesenchymal transition by acting as a co-activator. Genome Biol. (2014) 15:513. 10.1186/s13059-014-0513-0 25517638 PMC4253621

[13] Ibrahim AA Schmithals C Kowarz E Koberle V Kakoschky B Pleli T. Hypoxia causes downregulation of dicer in hepatocellular carcinoma, which is required for upregulation of hypoxia-inducible factor 1alpha and epithelial-mesenchymal transition. Clin Cancer Res. (2017) 23:3896–905. 10.1158/1078-0432.CCR-16-1762 28167508

[14] Stein JP Lieskovsky G Cote R Groshen S Feng AC Boyd S. Radical cystectomy in the treatment of invasive bladder cancer: long-term results in 1,054 patients. J Clin Oncol. (2001) 19:666–75. 10.1200/JCO.2001.19.3.666 11157016

[15] Babjuk M Bohle A Burger M Capoun O Cohen D Comperat EM EAU guidelines on non-muscle-invasive urothelial carcinoma of the bladder: update 2016. Eur Urol. (2017) 71:447–61. 10.1016/j.eururo.2016.05.041 27324428

[16] Wang L Saci A Szabo PM Chasalow SD Castillo-Martin M Domingo-Domenech J. EMT- and stroma-related gene expression and resistance to PD-1 blockade in urothelial cancer. Nat Commun. (2018) 9:3503. 10.1038/s41467-018-05992-x 30158554 PMC6115401

[17] Dongre A Weinberg RA. New insights into the mechanisms of epithelial-mesenchymal transition and implications for cancer. Nat Rev Mol Cell Biol. (2019) 20:69–84. 10.1038/s41580-018-0080-4 30459476

[18] Nieto MA Huang RY Jackson RA Thiery JP. Emt: 2016. Cell. (2016) 166:21–45. 10.1016/j.cell.2016.06.028 27368099

[19] Pastushenko I Brisebarre A Sifrim A Fioramonti M Revenco T Boumahdi S. Identification of the tumour transition states occurring during EMT. Nature. (2018) 556:463–8. 10.1038/s41586-018-0040-3 29670281

[20] van Kampen JGM van Hooij O Jansen CF Smit FP van Noort PI Schultz I. miRNA-520f reverses epithelial-to-mesenchymal transition by targeting ADAM9 and TGFBR2. Cancer Res. (2017) 77:2008–17. 10.1158/0008-5472.CAN-16-2609 28209612

[21] Yu C Liu Z Chen Q Li Y Jiang L Zhang Z. Nkx2.8 inhibits epithelial-mesenchymal transition in bladder urothelial carcinoma via transcriptional repression of *Twist1*. Cancer Res. (2018) 78:1241–52. 10.1158/0008-5472.CAN-17-1545 29311157

[22] Cheng X Wang X Wu Z Tan S Zhu T Ding K. CORO1C expression is associated with poor survival rates in gastric cancer and promotes metastasis *in vitro*. FEBS Open Bio. (2019) 9:1097–108. 10.1002/2211-5463.12639 30974047 PMC6551501

[23] Larzabal L Nguewa PA Pio R Blanco D Sanchez B Rodriguez MJ Overexpression of TMPRSS4 in non-small cell lung cancer is associated with poor prognosis in patients with squamous histology. Br J Cancer. (2011) 105:1608–14. 10.1038/bjc.2011.432 22067904 PMC3242532

[24] Chikaishi Y Uramoto H Koyanagi Y Yamada S Yano S Tanaka F. TMPRSS4 expression as a marker of recurrence in patients with lung cancer. Anticancer Res. (2016) 36:121–7. 26722035

[25] Villalba M Diaz-Lagares A Redrado M de Aberasturi AL Segura V Bodegas ME. Epigenetic alterations leading to TMPRSS4 promoter hypomethylation and protein overexpression predict poor prognosis in squamous lung cancer patients. Oncotarget. (2016) 7:22752–69. 10.18632/oncotarget.8045 26989022 PMC5008398

[26] Li XM Liu WL Chen X Wang YW Shi DB Zhang H. Overexpression of TMPRSS4 promotes tumor proliferation and aggressiveness in breast cancer. Int J Mol Med. (2017) 39:927–35. 10.3892/ijmm.2017.2893 28259959 PMC5360421

[27] Huang A Zhou H Zhao H Quan Y Feng B Zheng M. High expression level of TMPRSS4 predicts adverse outcomes of colorectal cancer patients. Med Oncol. (2013) 30:712. 10.1007/s12032-013-0712-7 24072509

[28] Guan H Liang W Liu J Wei G Li H Xiu L. Transmembrane protease serine 4 promotes thyroid cancer proliferation via CREB phosphorylation. Thyroid. (2015) 25:85–94. 10.1089/thy.2014.0155 25244400 PMC4290798

[29] Jin J Shen X Chen L Bao LW Zhu LM. TMPRSS4 promotes invasiveness of human gastric cancer cells through activation of NF-kappaB/MMP-9 signaling. Biomed Pharmacother. (2016) 77:30–6. 10.1016/j.biopha.2015.11.002 26796262

[30] Kim MS Kuppireddy SV Sakamuri S Singal M Getnet D Harsha HC. Rapid characterization of candidate biomarkers for pancreatic cancer using cell microarrays (CMAs). J Proteome Res. (2012) 11:5556–63. 10.1021/pr300483r 22985314 PMC3565537

[31] Cheng Y Wang K Geng L Sun J Xu W Liu D. Identification of candidate diagnostic and prognostic biomarkers for pancreatic carcinoma. EBioMedicine. (2019) 40:382–93. 10.1016/j.ebiom.2019.01.003 30639415 PMC6412825

[32] Jianwei Z Qi L Quanquan X Tianen W Qingwei W. TMPRSS4 upregulates TWIST1 expression through STAT3 activation to induce prostate cancer cell migration. Pathol Oncol Res. (2018) 24:251–7. 10.1007/s12253-017-0237-z 28466252

[33] Wang J Tsouko E Jonsson P Bergh J Hartman J Aydogdu E. miR-206 inhibits cell migration through direct targeting of the actin-binding protein coronin 1C in triple-negative breast cancer. Mol Oncol. (2014) 8:1690–702. 10.1016/j.molonc.2014.07.006 25074552 PMC5528580

[34] Lim JP Shyamasundar S Gunaratne J Scully OJ Matsumoto K Bay BH. YBX1 gene silencing inhibits migratory and invasive potential via CORO1C in breast cancer *in vitro*. BMC Cancer. (2017) 17:201. 10.1186/s12885-017-3187-7 28302118 PMC5356414

[35] Mataki H Enokida H Chiyomaru T Mizuno K Matsushita R Goto Y. Downregulation of the microRNA-1/133a cluster enhances cancer cell migration and invasion in lung-squamous cell carcinoma via regulation of Coronin1C. J Hum Genet. (2015) 60:53–61. 10.1038/jhg.2014.111 25518741

[36] McCarthy N. Tumour microenvironment: the same, but different. Nat Rev Cancer. (2011) 11:232. 10.1038/nrc3045 21548395

[37] Mantovani A. Cancer: inflaming metastasis. Nature. (2009) 457:36–7. 10.1038/457036b 19122629

[38] Huh SJ Liang S Sharma A Dong C Robertson GP. Transiently entrapped circulating tumor cells interact with neutrophils to facilitate lung metastasis development. Cancer Res. (2010) 70:6071–82. 10.1158/0008-5472.CAN-09-4442 20610626 PMC2905495

[39] Cools-Lartigue J Spicer J McDonald B Gowing S Chow S Giannias B. Neutrophil extracellular traps sequester circulating tumor cells and promote metastasis. J Clin Invest. (2013) 123:3446–58. 10.1172/JCI67484 23863628 PMC3726160

[40] Gaida MM Steffen TG Gunther F Tschaharganeh DF Felix K Bergmann F. Polymorphonuclear neutrophils promote dyshesion of tumor cells and elastase-mediated degradation of E-cadherin in pancreatic tumors. Eur J Immunol. (2012) 42:3369–80. 10.1002/eji.201242628 23001948

[41] Fang LY Izumi K Lai KP Liang L Li L Miyamoto H. Infiltrating macrophages promote prostate tumorigenesis via modulating androgen receptor-mediated CCL4-STAT3 signaling. Cancer Res. (2013) 73:5633–46. 10.1158/0008-5472.CAN-12-3228 23878190 PMC3833080

[42] Li S Cong X Gao H Lan X Li Z Wang W Tumor-associated neutrophils induce EMT by IL-17a to promote migration and invasion in gastric cancer cells. J Exp Clin Cancer Res. (2019) 38:6 10.1186/s13046-019-1168-1 30616627 PMC6323742

[43] Fan L Wei Y Ding X Li B. Coronin3 promotes nasopharyngeal carcinoma migration and invasion by induction of epithelial-to-mesenchymal transition. Onco Targets Ther. (2019) 12:9585–98. 10.2147/OTT.S215674 32009795 PMC6859123

[44] Yu G Wang LG Han Y He QY. clusterProfiler: an R package for comparing biological themes among gene clusters. OMICS. (2012) 16:284–7. 10.1089/omi.2011.0118 22455463 PMC3339379

[45] Hanzelmann S Castelo R Guinney J. GSVA: gene set variation analysis for microarray and RNA-seq data. BMC Bioinformatics. (2013) 14:7. 10.1186/1471-2105-14-7 23323831 PMC3618321

[46] Li B Severson E Pignon JC Zhao H Li T Novak J. Comprehensive analyses of tumor immunity: implications for cancer immunotherapy. Genome Biol. (2016) 17:174. 10.1186/s13059-016-1028-7 27549193 PMC4993001

[47] Li T Fan J Wang B Traugh N Chen Q Liu JS. TIMER: a web server for comprehensive analysis of tumor-infiltrating immune cells. Cancer Res. (2017) 77:e108–10. 10.1158/0008-5472.CAN-17-0307 29092952 PMC6042652

[48] Bindea G Mlecnik B Tosolini M Kirilovsky A Waldner M Obenauf AC. Spatiotemporal dynamics of intratumoral immune cells reveal the immune landscape in human cancer. Immunity. (2013) 39:782–95. 10.1016/j.immuni.2013.10.003 24138885

[49] Cerami E Gao J Dogrusoz U Gross BE Sumer SO Aksoy BA. The cBio cancer genomics portal: an open platform for exploring multidimensional cancer genomics data. Cancer Discov. (2012) 2:401–4. 10.1158/2159-8290.CD-12-0095 22588877 PMC3956037

