# Supplementary figures and images for: Novel Potential Biomarkers Associated With Epithelial to Mesenchymal Transition and Bladder Cancer Prognosis Identified by Integrated Bioinformatic Analysis
Source: Front Oncol. 2020 Jun 30;10:931. doi: 10.3389/fonc.2020.00931 (PMC7338771; doi:10.3389/fonc.2020.00931)

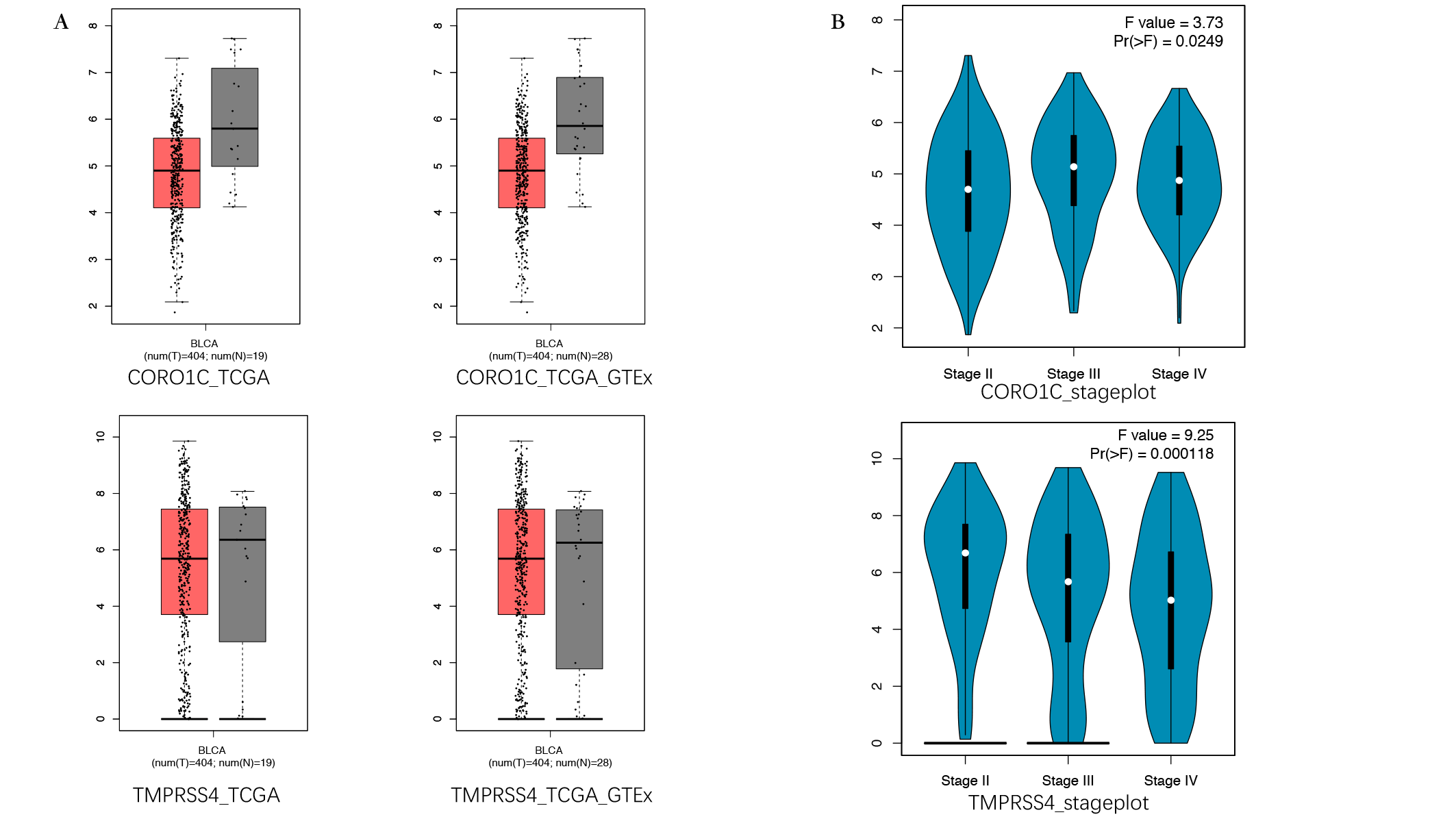

Supplement: Supplementary Figure 1 — (A) The expression of CORO1C and TMPRSS4 in normal bladder tissues and bladder cancer tissues in the TCGA and GTEx database. (B) The relationship between two hub genes and tumor stage via GEPIA online tool. [file Image_1_v1.TIF]
